# Correction: Network pharmacology and experimental evaluation of Sanwei Tanxiang Tangsan in doxorubicin-induced heart failure

**DOI:** 10.1186/s13019-026-04682-w

**Published:** 2026-09-28

**Authors:** Aruna, Dulan, Davaadagva Damdinjav, Battulga Tungalag, GuiZhi, Hua Li

**Affiliations:** 1 Inner Mongolia Medical University, Hohhot City, 010110 Inner Mongolia Autonomous Region China; 2 Mongolian National University of Medical Sciences, Ulaanbaatar, 999097 Mongolia

**Correction: Journal of Cardiothoracic Surgery (2026) 21:581**

**https://doi.org/10.1186/s13019-026-04362-9**

The Author Contributions section of this article [1] contains incorrect author names and initials that need to be updated as follows:

Lan Du (LD) should be corrected to Dulan (DL).

Zhi Gui (ZG) should be corrected to GuiZhi (GZ).

AR and LD should be corrected to AR and DL.

AR and ZG should be corrected to AR and GZ.

Updated Author contributions is given below:

**Author contributions**

ARuNa (AR), Dulan (DL), and GuiZhi (GZ) gathered data and analyzed and interpreted it. AR and DL performed the network pharmacology and molecular docking analyses. AR and GZ carried out the animal experiments. AR and Hua Li (HL) conceptualized and designed the research. HL supervised this work, gave critical comments on the research, revised the intellectual content, and edited the final manuscript. Davaadagva Damdinjav (DD) and Battulga Tungalag (BT) contributed to data interpretation and manuscript revision. All authors read and agreed to the published version of the manuscript.

The original article has been corrected.
